# MVP expression in the prediction of clinical outcome of locally advanced oral squamous cell carcinoma patients treated with radiotherapy

**DOI:** 10.1186/1748-717X-7-147

**Published:** 2012-08-29

**Authors:** Luis Alberto Henríquez-Hernández, Mercedes Moreno, Agustín Rey, Marta Lloret, Pedro C Lara

**Affiliations:** 1 Radiation Oncology Department, Hospital Universitario de Gran Canaria Dr. Negrín, Barranco de La Ballena s/n., Las Palmas de Gran Canaria, CP 35010, Spain; 2 Instituto Canario de Investigación del Cáncer (ICIC), Las Palmas de Gran Canaria, Spain; 3 Clinical Sciences Department, Universidad de Las Palmas de Gran Canaria; 4 Department of Maxillofacial Surgery, Hospital Universitario de Gran Canaria Dr. Negrín, Las Palmas de Gran Canaria, Spain; 5 Department of Pathology, Hospital Universitario de Gran Canaria Dr. Negrín, Las Palmas de Gran Canaria, Spain

**Keywords:** MVP, IGF-1R, Oral carcinoma, Radiotherapy, Predictive factor

## Abstract

**Objective:**

To explore the role of Major Vault Protein (MVP) in oral cavity squamous cell carcinoma patients.

**Subjects and Methods:**

131 consecutive patients suffering from oral cavity squamous cell carcinoma were included in the study. In the whole series, the mean follow-up for survivors was 123.11 ± 40.36 months. Patients in tumour stages I and II were referred to surgery; patients in stage III-IV to postoperative radiotherapy (mean dose = 62.13 ± 7.74 Gy in 1.8–2 Gy/fraction). MVP expression was studied by immunohistochemistry in paraffin-embedded tumour tissue.

**Results:**

MVP expression was positive in 112 patients (85.5%) and no relation was found with clinic pathological variables. MVP overexpression (those tumours with moderate or strong expression of the protein) was related to insulin-like growth factor receptor-1 (IGF-1R) expression (P = 0.014). Tumour stage of the disease was the most important prognostic factor related to survival. Tumours overexpressing MVP and IGF-1R were strongly related to poor disease-free survival (P = 0.008, Exp(B) = 2.730, CI95% (1.302-5.724)) and cause-specific survival (P = 0.014, Exp(B) = 2.570, CI95% (1.215-5.437)) in patients achieving tumour stages III-IV, in multivariate analysis.

**Conclusions:**

MVP and IGF-1R expression were related in oral squamous cell carcinoma and conferred reduced long-term survival in patients suffering from advanced stages of the disease.

## Background

Oral carcinoma is commonly treated by surgery or radiotherapy as local treatments. Although long-term survival is improving with advances in therapy, outcomes in locally advanced cases remain suboptimal. There is a clear need for new prognostic indicators, which could be used in diagnostics and, consequently, in the selection of the most effective treatment method.

Vaults are ribonucleoprotein particles with a hollow barrel-like structure composed of three proteins (the 110 kDa major vault protein (MVP), the two minor vault poly(ADP-ribose) polymerase (VPARP), and the 240 kDa telomerase-associated protein-1 (TEP1)) and small untranslated RNA (vRNA) [1]. Vaults have been classically associated to multidrug resistance [2]. However, it has been pointed that MVP interacts with several proteins involved in relevant cellular mechanisms as PTEN, Erk, or EGF. Furthermore, the expression of MVP was associated to a malignant phenotype in some cancers, indicating a direct involvement in tumour development and progression [3]. MVP has been associated to resistance to radiotherapy [4], probably due to its role in preventing apoptosis by inhibiting the COP-1/p53 axis [5]. Various DNA damaging agents, including ultraviolet irradiation, induce increased MVP transcription and protein levels [6]. This suggests that vaults may have a role in facilitating DNA repair processes, which is consistent with previous work showing that VPARP- and TEP1-deficient mice have an increased incidence of carcinogen-induced colon tumours [7]. At the clinical level, the role of MVP in predicting response to radiotherapy was recently first addressed [8]. In that study, MVP was related to poor outcome after radiotherapy in 78 patients suffering from oropharyngeal carcinoma. The estimated effects of MVP overexpression appeared somewhat larger in the tongue cancer patients compared with the tonsil cancer patients for loco-regional failure and cancer-specific death [8]. However, the underlying mechanisms behind this observation are not understood and the role of MVP in oral cavity squamous cell carcinoma has not been deeply studied, especially in combination with other biological markers.

Insulin-like growth factor-1 receptor (IGF-1R) is a transmembrane tyrosine kinase receptor commonly overexpressed in many cancers. Activation of IGF-1R leads to activation of the ras, raf and MAPK pathways, resulting in increased proliferation; and of the PI3K pathway which in turn results in the prevention of apoptosis. IGF-1R activation has been associated with increased radioresistance by increasing cell proliferation and prevention of apoptosis [9,10]. The expression of IGF-1R directly influences radioresistance [11]. In that sense, we have previously reported that IGF-1R overexpression is associated with reduced long-term local control in cervical [12] and oral cancer patients [13]. An association between MVP and IGF-1R expression has been reported in cervical patients [14]. Thus, the combined overexpression of MVP and IGF-1R conferred reduced long-term survival in patients suffering from cervical cancer who achieved clinical complete response to radiochemotherapy [14].

The aim of the present study was to assess the expression of MVP in oral cavity squamous cell carcinoma patients, its relation with clinical and pathologic prognostic factors and its role in predicting clinical outcome. In addition, we explored the relation to IGF-1R expression in this cohort of patients.

## Materials and Methods

### Patients

The present series of patients was collected from the tumour registry of the Maxillofacial Surgery Department of our Institution. All patients were diagnosed and treated between July 1989 and April 2005. Cases were excluded from this study if i) they were diagnosed in other Institution, ii) if pathology blocks were not available, iii) or if patients received any kind of chemotherapy either pre or post surgery. Thus, one hundred and thirty one patients suffering from oral cavity squamous cell carcinoma (OCSCC) were included in this study. Patients were diagnosed and treated by surgery and curative radiation therapy at the Hospital Universitario de Gran Canaria Dr. Negrín (Las Palmas de Gran Canaria, Spain). All patients included in the study received and signed an informed consent. The study was approved by the Research Committee of our Institution. Follow-up was closed in July 2011. The mean follow-up for survivors (n = 18, 15 males and 3 females) was 123.11 ± 40.36 months (median 113.50, range 72–204 months). Patients were staged following the TNM classification and grading according to Broders’ system. Nineteen patients had stage I disease, 46 stage II, 25 stage III and 41 stage IV. Most patients showed tongue tumours (64 cases) and grades I (39 cases) and II (74) carcinomas. Characteristics of patients were summarized in Table 1.

**Table 1:** Characteristics of patients in study (n = 131)

**Characteristic**	**N**	**(%)**
**Gender**		
Male	116	(88.5)
Female	15	(11.5)
**Age** (years)		
<59	62	(47.3)
>59	69	(52.7)
**Stage**		
I	19	(14.5)
II	46	(35.1)
III	25	(19.1)
IV	41	(31.3)
**Grade**		
I	39	(29.8)
II	74	(56.5)
III	18	(13.7)
**Treatment**		
Surgery + Radiotherapy	84	(64.1)
Surgery	131	(100)
**Surgery margins**		
Affected	44	(33.6)
Non-affected	87	(66.4)

### Treatment

All patients were referred to surgery. Additionally, 14 patients were referred to functional cervical dissection, 102 patients were referred to radical cervical dissection and 1 patient was referred to bilateral cervical dissection. Fourteen patients did not suffer neck dissection. Those patients in stages III-IV were referred to postoperative radiotherapy (RT). Patients in stages I and II were referred to RT if tumour positive margins were found at surgery. Thus, 84 patients received postoperative RT up to a mean dose of 62.13 ± 7.74 Gy (median 65 Gy, range 30–70.20 Gy) in 1.8–2 Gy fractions. No patients were treated with adjuvant or neoadjuvant chemotherapy.

### Immunohistochemistry

MVP expression was studied by immunohistochemistry in all patients as previously described [14,15]. In short, sections from paraffin-embedded tissue tumour biopsies were incubated with mouse anti-MVP monoclonal antibody (LRP/MVP Ab-2, Clon 1032, Neomarkers, CA) applied at a 1:100 dilution and incubated for 1 hour at room temperature in a moist chamber. A secondary biotinylated antibody (DAKO Detection Kit, LSBA, Carpintería, CA) and a prepared complex peroxidase–streptavidin biotinylated (DAKO, LSBA, Carpintería, CA) were used afterwards. Staining was revealed (DAKO, DAB Chromogen, Carpintería, CA) and counterstained with Harris hematoxylin. The primary antibody was omitted in one section as a negative control and a strongly positive tumour for MVP was used as a positive control.

**Figure 1:**
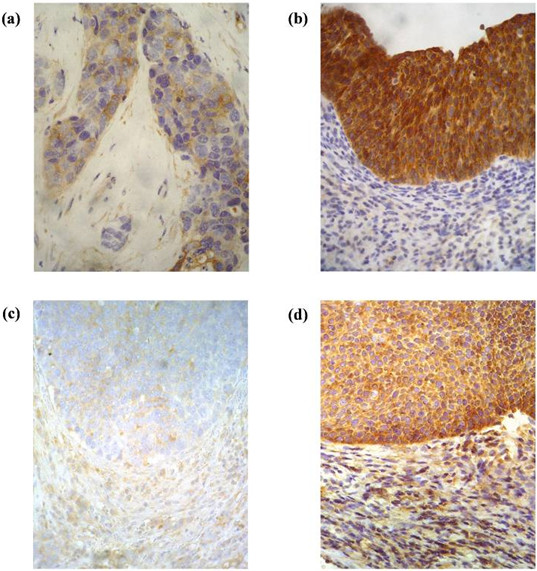
Representative immunostaining of IGF-1R and MVP scored semi quantitatively (400x), showing (a, c) low and (b, d) overexpression, respectively.

MVP staining, observed in the cell cytoplasm, was estimated blindly of the clinical patient characteristics and outcome in zones of maximum expression of the marker in at least 10 high power fields (400×). MVP expression was scored semi quantitatively taking into account intensity and area of staining; and classified as low expressed (negative (−) or slightly (+) positive) or overexpressed (moderately (++) or strongly (+++) positive) as previously reported [14] (Figure 1). Data on IGF-1R expression were obtained from our files [13] following the same protocol for staining and scoring as previously described. 

### Statistical analysis

Outcome after treatment was assessed as Local Disease Free Survival (LDFS), Regional Disease Free Survival (RDFS), Distant-Metastasis Free Survival (DMFS), Disease-Free Survival (DFS), and Cause-Specific Survival (CSS). Correlations between previously described variables and outcome during follow-up were analyzed by the Kaplan–Meier survival test and the differences were calculated by the log-rank test. Cox regression test was used for multivariate analysis. Statistical analysis was performed by SPSS 15.0 software.

## Results

MVP and IGF-1R expression was established for all 131 cases. MVP expression was positive in 112 patients (85.5%) and it was overexpressed in 67 patients (51.1%). IGF-1R was overexpressed in 60 patients (45.8%). The expression of both oncoproteins was statistically different among patients (P = 0.014, Table 2). We observed a positive association between the expression of MVP and IGF-1R (Pearson correlation coefficient = 0.224, P = 0.010). We did not find other statistical associations between other variables studied.

**Table 2:** Relation of MVP and IGF-1R expression in oral tumours

	**MVP**		
	**Low expression**	**Over expression**	**Total**
IGF-1R Low expression	42	29	71
Over expression	22	38	60
Total	64	67	131

Expression of oncoproteins was scored semi quantitatively and classified as low (negative/slightly positive) or overexpressed (moderately/strongly positive). Chi square test, P = 0.014.

Actuarial survival ratios after 15 years of follow-up in the whole series were 65.6, 70.2, 90.6, 49.6, and 57.1% for LDFS, RDFS, DMFS, DFS, and CSS, respectively. Tumour grade (I vs. II-III) and stage (I-II vs. III-IV) were the most important prognostic factors for CSS in multivariate analysis (Exp(B) = 3.009, confidence interval [CI] 95% (1.255-7.214), P = 0.014; Exp(B) = 3.085, CI95% (1.565-6.083), P = 0.001, respectively). MVP and IGF-1R expression were not differentially expressed according to tumour location and were not predictive factors in the whole series (Data not shown).

Because tumour stage was the most important prognostic factor for survival, we analyzed the role of MVP on long-term control and survival segmenting patients according to the pathological tumour stage. Twenty out of the 30 patients (66.7%) suffering tumour stages III-IV and negative or fairly positive MVP expression were alive, while 12 out of the 36 patients (33.3%) with tumour stages III-IV and overexpression of MVP were alive. The results of log rank tests were statistically significant for DMFS (P = 0.030) and DFS (P = 0.020) (Table 3). Moreover, MVP overexpression was a predictive factor for DFS (P = 0.016, Exp(B) = 2.584, CI95% (1.191-5.604)) and CSS (P = 0.011, Exp(B) = 2.916, CI95% (1.286-6.629)) in multivariate analysis. MVP expression was not a predictive factor in patients suffering stage I-II of the disease. Stratified analyses by tumour grade and surgery margins did not show any association with MVP expression (P > 0.05 in all cases).

**Table 3:** Relation of prognostic variables and clinical outcome in locally advanced oral carcinoma patients (stages III-IV), n = 66

	**LDFS**	**RDFS**	**DMFS**	**DFS**	**CSS**
**Gender**	P = 0.683	P = 0.744	P = 0.524	P = 0.707	P = 0.485
**Age**	P = 0.882	P = 0.537	P = 0.387	P = 0.574	P = 0.866
**Grade**	P = 0.330	P = 0.053	P = 0.047	P = 0.024	P = 0.008
**Surgery margins**	P = 0.001	P = 0.077	P = 0.388	P = 0.003	P = 0.001
**MVP**	P = 0.214	P = 0.362	P = 0.030	P = 0.020	P = 0.010
**IGF-1R**	P = 0.016	P = 0.153	P = 0.898	P = 0.029	P = 0.009

P values obtained by log rank test.

**Figure 2:**
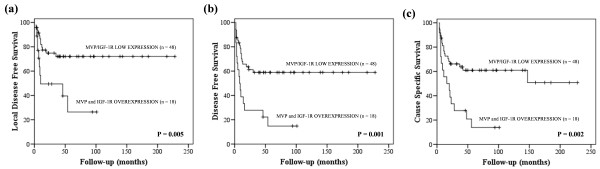
**MVP/IGF-1R expression and clinical outcome in oral carcinoma patients suffering from tumour stages III-IV (n = 66).** (**a**) Local Disease Free Survival, (**b**) Disease-Free Survival, and (**c**) Cause-Specific Surviva.

Combination analysis was performed grouping MVP and IGF-1R expression in tumours. These analyses showed that 29 out of the 48 patients (60.4%) suffering from tumour stages III-IV and negative or fairly positive MVP/IGF-1R expression were alive, while 3 out of the 18 patients (16.7%) with tumour stages III-IV and overexpression of MVP and IGF-1R were alive (P = 0.002). The results of log rank tests were also statistically significant for LDFS (P = 0.005) and DFS (P = 0.001) (Figure 2a-c). In multivariate analysis, MVP and IGF-1R overexpression was a predictive factor for DFS (P = 0.008, Exp(B) = 2.730, CI95% (1.302-5.724)) and CSS (P = 0.014, Exp(B) = 2.570, CI95% (1.215-5.437)).

## Discussion

This study was carried out to explore the clinical association between MVP expression and prognosis on a series of OCSCC patients treated by surgery and radiotherapy. MVP was overexpressed in 67 (51.1%) of our oral cavity carcinoma patients. Only one study has reported data of MVP expression in head and neck cancer [8]. Silva et al. detected positive expression of MVP in 49 out of 78 (62.8%) patients suffering from carcinoma in the posterior third of tongue and the tonsil.

Tumour stage was the most important prognostic factor for survival in our series. The rates of local recurrence for clinical stages III or IV tumours may be as high as 40%. In our whole series, MVP was not a predictive factor for survival. Nonetheless, when patients were segregated according to low (I-II) or high (III-IV) tumour stage, MVP expression was predictive for DMFS, DFS, and CSS. The fact that patients with advanced stages of the disease suffer more amount of adverse events related to the tumour, together with the radioresistance role of MVP previously reported in tongue and cervical carcinoma, helps to explain the predictive role of this oncoprotein only in patients suffering OCSCC in stage III-IV, all treated with surgery and RT. MVP may have a role in favouring increased genetic instability by reducing DNA damage repair by means of non-homologous end joining (NHEJ) and downregulating Ku70/80 expression [16]. The most important radiation-induced DNA damage is the double-strand breaks (DSB), mainly repaired by NHEJ. Ku70/80 are key genes in the NHEJ repair pathway for radiation-induced DNA DSB. If vaults are overexpressed, NHEJ repair may be suppressed by several mechanisms; thus, genomic instability could arise because either repair of DSB damage by homologous repair is prevented or NHEJ repair is defective. The PARP-1, part of Vault complex, promotes homologous repair over NHEJ, suppressing Ku70/80 function. These events are associated with the decision of damaged cells to survive and proliferate, favouring tumour progression and reducing tumour response to oncologic treatment through the development of resistant cell phenotypes. Our results are in agreement with the published study of Silva et al. [8] where MVP has strongly predicted the clinical outcome in 78 oropharyngeal cancer patients who received primary radiotherapy with a curative intent. Because OCSCC in advanced stages (III-IV) were all referred to postoperative radiotherapy, the role of MVP was only observed in this subset of patients and not in the whole series.

We reported here the first clinical evidence of a direct relation between IGF-1R and MVP expression in OCSCC. The association between both oncoproteins has been previously reported in cervical carcinoma patients [14]. It seems that both proteins must be overexpressed to confer radioresistance and subsequent poor clinical outcome in cervical cancer. It would be suggested that both MVP and IGF-1R increase proliferation by activation of Src, PTEN, ERK and inhibit apoptosis through the ATM COP1/P53 axis, resulting in tumour resistance to DNA damage agents. It has been reported an association between MVP expression and cell proliferation, as well as other molecules involved in apoptosis [16]. Thus, resistance to radiotherapy in the clinical setting as a result of MVP [8,14] may be causes by the enhanced of proliferation and inhibition of apoptosis, as well as by the regulation of DSB repair by suppressing NHEJ repair through the inhibition of Ku70/80 [16]. The IGF-1R cooperates with MVP in preventing apoptosis by upregulating BCL-2 and, to a lesser extent, downregulating BAX [16], and it may also affect NHEJ repair through the AKT/p38MAPK pathways, mainly by modifying Ku expression [17]. Activation of IGF-1R triggers a cascade of reactions involving signal transduction pathways: Ras, Raf, mitogen-activated protein kinase and phosphoinositol-3-kinase (PI3K)/AKT/BAD (Bcl-xL/Bcl2-associated death promoter). IGF-1R overexpression has been linked to disease progression, increased resistance to radiotherapy, and poor prognosis [9,12,18]. IGF-1R gene seems to be a novel downstream target in an Ataxia Telangiectasia Mutated (ATM)-mediated DNA damage response pathway. The potential role of the IGF-1R gene as a target ing an ATM-dependent pathway, involved in regulating the radiation response, was recently inferred [19]. Thus, deregulated expression of the IGF-1R gene after ionizing radiation may be linked to genomic instability and enhanced transforming capacity [20].

The expression of MVP has a complex regulation not fully understood, influenced by the expression of other oncoproteins and other important clinical factors as hypoxia. In that sense, high MVP expression is related to severe hypoxia in clinical cervical tumours [21]. Interestingly, hypoxia inhibits the NHEJ DNA repair through down-regulating Ku70/80 expression, combined with increased angiogenesis and altered p53 [22], and an inverse association between MVP and KU70/80 exists [16]. Although the prognosis role of MVP is still contradictory, at a clinical level, vaults have been proposed as a useful predictive marker associated with radiotherapy resistance [23], and the interaction between IGF-1R and MVP can help to explain the predictive role of these oncoproteins in response to radiotherapy [24].

## Conclusions

MVP and IGF-1R expression were related in OCSCC tumours and conferred reduced long-term survival in patients suffering advanced stages of the disease. The present results could help to understand the biological behavior of this tumor type according to the expression not only of IGF-1R, but also of MVP. In our opinion, combined MVP/IGF-1R expression could be of relevance in predicting the clinical outcome in this tumour type. However, new studies including PTEN, P53, BCL-2, cell proliferation, hypoxia or PI3K markers would be necessary to precisely define the role of these proteins on common molecular pathways. These preliminary results need to be confirmed by a larger clinical trial.

## Abbreviations

ATM: Ataxia Telangiectasia Mutated; DFS: Disease-Free Survival; DMFS: Distant-Metastasis Free Survival; DSB: Double-strand break; IGF-1R: Insulin-like growth factor-1 receptor; LDFS: Local Disease Free Survival; MVP: Major Vault Protein; NHEJ: Non-homologous end-joining; OCSCC: Oral cavity squamous cell carcinoma; RDFS: Regional Disease Free Survival.

## Competing interests

There is no competing interest for all authors.

## Authors’ contributions

LAHH has made analysis and interpretation of data and has been involved in drafting the manuscript; MM and AR have been involved in acquisition of data; MLl has been involved in conception and design of the study and revised the manuscript critically; PCL has been involved in conception and design of the study, has contributed to analysis and interpretation of data and has given final approval of the version to be published.
